# Combined anterior lumbar interbody fusion and instrumented posterolateral fusion for degenerative lumbar scoliosis: indication and surgical outcomes

**DOI:** 10.1186/s12893-015-0006-4

**Published:** 2015-03-15

**Authors:** Ming-Kai Hsieh, Lih-Huei Chen, Chi-Chien Niu, Tsai-Sheng Fu, Po-Liang Lai, Wen-Jer Chen

**Affiliations:** Department of Orthopedic Surgery, Chang Gung Memorial Hospital and Chang Gung University, 5, Fu-Hsin Street, Kweishan Shiang, Taoyuan, 333 Taiwan

**Keywords:** Degenerative lumbar scoliosis, De novo scoliosis, Anterior lumbar interbody fusion, Instrumented fusion

## Abstract

**Background:**

Traditional approaches to deformity correction of degenerative lumbar scoliosis include anterior-posterior approaches and posterior-only approaches. Most patients are treated with posterior-only approaches because the high complication rate of anterior approach. Our purpose is to compare and assess outcomes of combined anterior lumbar interbody fusion and instrumented posterolateral fusion with posterior alone approach for degenerative lumbar scoliosis with spinal stenosis.

**Methods:**

Between November 2002 and November 2011, a total of 110 patients with degenerative spinal deformity and curves measuring over 30°were included. Of the 110 patients who underwent surgery, 56 underwent the combined anterior and posterior approach and 54 underwent posterior surgery at our institution. The following were the indications of anterior lumbar interbody fusion: (1) rigid or frank lumbar kyphosis, (2) anterior or lateral bridged traction osteophytes, (3) gross coronal and sagittal deformity or imbalance, and (4) severe disc space narrowing that is not identifiable when performing posterior or transforaminal lumbar interbody fusion. The clinical outcomes were evaluated using the Oswestry disability index and the visual analog scale. The status of fusion were assessed according to the radiographic findings.

**Results:**

All patients received clinical and radiographic follow-up for a minimum of 24 months, with an average follow-up of 53 months (range, 26–96 months). At the final follow-up, the mean ODI score improved from 28.8 to 6.4, and the mean back/leg VAS, from 8.2/5.5 to 2.1/0.9 in AP group and the mean ODI score improved from 29.1 to 6.2, and the mean back/leg VAS, from 9.0/6.5 to 2.3/0.5 in P group. The mean scoliotic angle changed from 41.3° preoperatively to 9.3°, and the lumbar lordotic angle, from 3.1° preoperatively to 35.7°in AP group and the mean scoliotic angle from 38.5 to 21.4 and the lumbar lordotic angle from 6 to 15.8 in P group. There were significant differences in sagittal (P = 0.009) and coronal (P = 0.02) plane correction between the two groups.

**Conclusions:**

Our results demonstrate that combined anterior lumbar interbody fusion and instrumented posterolateral fusion for adult degenerative lumbar scoliosis effectively improves sagittal and coronal plane alignment than posterior group and both group were effectively improves clinical scores.

## Background

Degenerative lumbar scoliosis is believed to develop secondary to asymmetric collapse of the intervertebral disc spaces [1-5]. This leads to poor body posture, back pain, and neurological deterioration owing to decreased foraminal height with nerve root compression on the concave side of the deformity, and nerve stretching on the convex side [1,2,6]. The commonly presenting symptoms include chronic back pain and neurogenic claudication caused by concurrent stenosis with a structural degenerative deformity [7]. Traditional approaches to deformity correction of degenerative lumbar scoliosis include anterior-posterior approaches and, more commonly, posterior-only approaches. Most patients are treated with posterior-only approaches because the anterior approach has been shown to be associated with complications such as vascular injury, ileus, and retrograde ejaculation and involves performing 2 large surgical procedures and, hence, increases the operating time [8,9]. The use of posterior decompression with posterior spinal instrumentation and fusion may obviate the need for extensive abdominal surgery by enabling significant correction through a posterior-only approach. However, degenerative lumbar scoliosis secondary to an idiopathic curve tends to become rigid anteriorly, which gets more difficult to be corrected via a posterior-only approach [10]. Combined anterior lumbar interbody fusion and instrumented posterolateral fusion provides several benefits over the posterior-only approach, in terms of improved stability, decreased stress on screws, improved fusion rates, and better lumbar lordosis [11-13]. To our knowledge, no study has yet mentioned the indications of combined anterior lumbar interbody fusion and instrumented posterolateral fusion for degenerative lumbar scoliosis with spinal stenosis and compare and assess outcomes with posterior alone approach.

The goals of adult deformity surgery are to obtain sagittal and coronal balance, symptom relief, and solid fusion [14,15]. Various techniques have been reported for correcting degenerative lumbar deformities with instrumentation and fusion using pedicle screw systems and various types of interbody cages. Interbody cages allow for the correction of the deformity, anterior column support, increased foraminal height, circumferential arthrodesis, and restoration of the anterior column height as well as lumbar lordosis. Interbody cages can be placed via either a posterior approach, as for the posterior lumbar interbody fusion (PLIF) and transforaminal lumbar interbody fusion (TLIF), or an anterior approach, as for the anterior lumbar interbody fusion (ALIF), by using either an autograft or allograft, metal cages, or poly-ether-ether-ketone (PEEK) cages [11,16-18]. Herein, we describe our experience as well as indications for performing combined anterior lumbar interbody fusion and instrumented posterolateral fusion for degenerative lumbar scoliosis with spinal stenosis.

## Methods

### Patients

**Figure 1:**
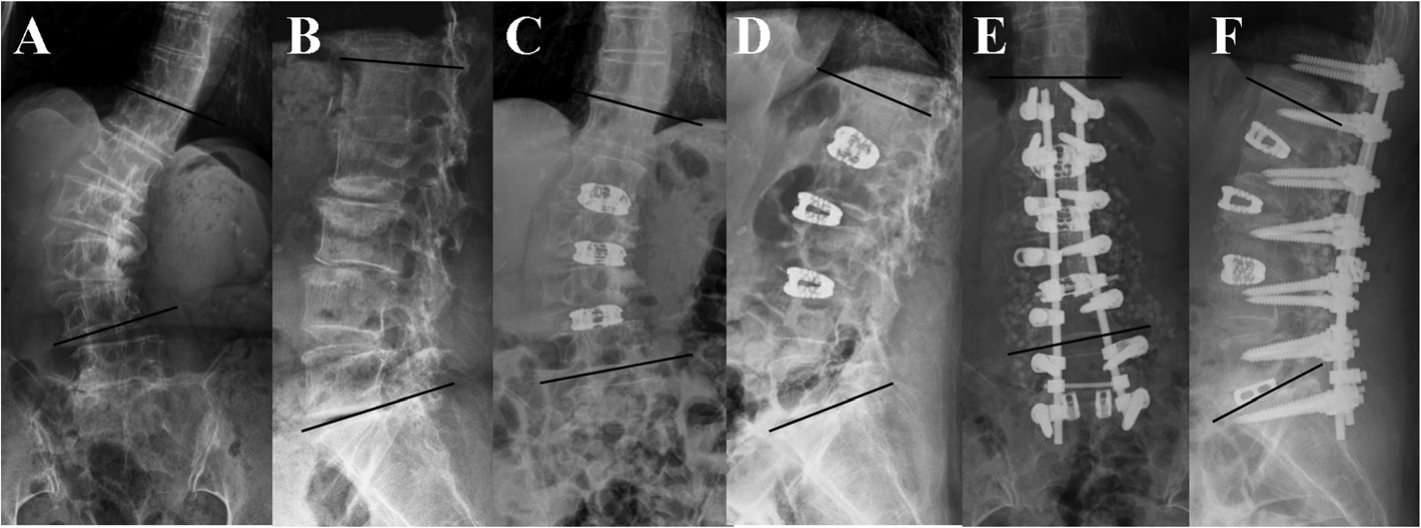
**A 64-year-old woman complained low back pain with bilateral sciatica and claudication for several years.** Radiographs of anteroposterior view **(A)** and lateral view **(B)** showing degenerative lumbar scoliosis from T12 to L5 with lateral bridged traction vertebral osteophytes over L2-3,L3-4 associated with severe disc space narrowing over L1-2 ,L2-3. After anterior lumbar interbody fusion with three SynCages over L1-2, L2-3, and L3-4, the scoliotic angle (T12-L4) was improved from 37° to 17° **(C)** and the lumbar lordotic curve was improved from 4° to 29° **(D)**. One week later, posterior instrumentation of T12-S1 with posterior interbody fusion of L5-S1 was performed. The scoliotic angle was improved from 17° to 6° **(E)** and the lumbar lordotic curve was improved from 29° to 36° **(F)**.

From November 2002 to November 2011, 1834 patients with degenerative lumbar scoliosis underwent surgery in our institution. The Chang Gung Medical Foundation Institutional Review Board approved this study (99-0771B) and waived the requirement for informed consent due to the retrospective nature of the study. All patients presented with neurological claudication with mechanical back pain that was refractory to at least 6 months of conservative management such as physical therapy, activity modification, chiropractic manipulation, administration of oral analgesics and nonsteroidal anti-inflammatory drugs, epidural steroids, and facet injections. The inclusion criteria of combined anterior and posterior approach were (1) rigid or frank lumbar kyphosis, (2) anterior or lateral bridged traction vertebral osteophytes, (3) gross coronal and sagittal deformity or imbalance, and (4) severe disc space narrowing that is not identifiable when performing PLIF or TLIF (Figure 1) and exclusion criteria were previous abdominal or retroperitoneal surgery.

A total of 110 patients with degenerative spinal deformity and curves measuring over 30°who underwent reconstructive spinal fusion surgery from 2002 to 2011 were included. Of the 110 patients who underwent surgery, 56 underwent the combined anterior and posterior approach and 54 underwent posterior surgery at our institution. This posterior (P) group included 34 females and 20 males with an average age of 62 years. 56 patients underwent combined anterior release and fusion of multiple lumbar levels followed by posterior instrumented fusion (AP group) included 35 females and 21 males with an average age of 61 years. Eighteen patients underwent ALIF followed by simultaneous instrumented posterolateral fusion. The remaining 38 patients underwent staged operations between 1 and 2 weeks.

### Clinical assessment

The clinical outcome was evaluated using the Oswestry disability index (ODI) and the visual analog scale (VAS) preoperatively and at the final follow-up. All patients were scheduled for follow-up at 3 months, 6 months, and 1 year after the surgery and then annually. The following comorbidities were preoperatively diagnosed in AP and P groups : diabetes mellitus (n = 6;8); hypertension (n = 8:8); corticosteroid usage (n = 3;4); and valvular heart disease (n = 2;1).

### Surgical technique

Anterior surgery in the combined anterior-posterior group, the patient was placed in the lateral decubitus position with the concave side up, with the intention to directly correct the scoliosis. ALIF was performed using the flank retroperitoneal approach. After exposure of the anterior part of the disc, the anterior longitudinal ligament was transversely incised, and the disc was completely removed. Next, the vertebral endplates were cleared of cartilage using sharp curettes, taking care that damage to the subchondral bone of the endplates is avoided. Maximum distraction of disc space was achieved by manual lordotic force. After a satisfactory trial implantation, the SynCage (Synthes Spine, West Chester, PA, USA), which was filled with a morselized cancellous allograft, was implanted.

Posterior surgery in the combined anterior-posterior group and the posterior group ,all posterior instrumentation was placed via an open posterior approach. After subperiosteal exposure of the dorsal spine using a standard midline approach, an autologous bone graft was harvested via a subcutaneous access to the posterior iliac crest. After adequate decompression, pedicle screw instrumentation with TriFix G (Aspine, Oakland, CA, USA), with or without PLIF or TLIF was performed. Finally, rod derotation maneuver and compression on the convex side with the rod carefully contoured to the lordosis was performed to restore lumbar lordosis and correct lumbar scoliosis.

### Radiographic evaluation

Plain radiographs in the standing posteroanterior (PA), lateral, and flexion-extension views were obtained for all patients preoperatively, postoperatively, 1 year after the surgery, and at the final follow-up. Preoperative and postoperative radiographs were compared to determine the degree of correction achieved following surgery. The coronal Cobb angle was determined from the standing PA radiograph by drawing a line parallel to the superior endplate of the most superior vertebra and a second line parallel to the inferior endplate of the most inferior vertebra of the scoliotic curve. Lumbar lordosis was measured using the Cobb method [18] between the superior endplate of L-1 and S-1. Hyperlordosis was defined as any Cobb angle >60°, and hypolordosis was defined as any angle <20°. We also measure the lordotic angle correction for each level and SynCage position. End-plate fractures, cage malpositioning, and the status of the anterior and posterolateral fusion were also recorded. Anterior fusion was classified as solid, probable, or pseudoarthrosis. Solid fusion was defined as visible, continuous trabeculae of bridging fusion masses across the disc space and lack of instability in the flexion-extension radiographs. Probable fusion was defined as unclear bony trabecular continuity with no radiolucent interruption or motion seen in the stress radiographs. Pseudoarthrosis was defined as radiolucent interruption of the cage, and as motion, in stress radiographs. Posterolateral fusion was also classified as solid, probable, and pseudoarthrosis. Solid fusion was defined as visible, continuous trabeculae of bridging fusion masses over the bilateral transverse processes and no motion in the flexion and extension stress radiographs. Probable fusion was defined as unclear bony trabecular continuity with no radiolucent interruption or motion in stress radiographs. Radiolucent interruption of the fusion mass was labeled as pseudoarthrosis [19]. The fusion status was decided by the senior surgeons (W-J,Chen ).

### Statistical analysis

Data were analyzed using the SPSS statistical software package (SPSS, Inc, Chicago, IL, USA). Means were calculated for different variables including the ODI score, VAS, and angles of lumbar lordosis and scoliosis. Preoperative and postoperative measurements and values between the different subgroups were compared using the paired *t*-test with statistical significance set at a P value of <0.05. The position of SynCage and the lordotic angle correction was compared using the Student’s *t*-test with statistical significance set at a P value of <0.05.

## Results

All patients received clinical and radiographic follow-up for a minimum of 24 months, with an average follow-up of 53 months (range, 26–96 months). The operation time of anterior lumbar interbody fusion is 172.5 minutes (range,115-301 minutes) and instrumented posterolateral fusion is 262.5 minutes (range, 195–375 minutes) in AP group and 350.5 minutes (range, 210–452 minutes) in P group, estimated blood loss of anterior lumbar interbody fusion is 250 ml (range,150-1750 ml) and instrumented posterolateral fusion is 1650 ml (range,1000-4850 ml) in AP group and 3250 ml (range,2000-6500 ml) in P group, transfusion amount of anterior lumbar interbody fusion is 700 ml (range,500-2000 ml) and instrumented posterolateral fusion is 1500 ml (range,1000-5000 ml) in AP group and 4000 ml (range,2000-8000 ml) in P group, and length of stay is 16 days (range ,10-21 days ) in AP group and 10 days (range ,7-14 days ) in P group.

### Clinical outcomes

The VAS and ODI scores were evaluated preoperatively and at the final follow-up (Table 1). At final follow-up, the average ODI score was significantly lower than that determined preoperatively in both groups. The mean back and leg VAS scores also improved significantly in both groups.

**Table 1:** **Clinical and radiographic outcomes**

	**AP** ^**#**^ **group**	**P** ^**##**^ **group**	**P value**
Pre-op mean back VAS^**+**^	8.2	9.0	0.54
2-year post-op mean back VAS	2.1	2.3	0.23
Pre-op mean leg VAS	5.5	6.5	0.45
2-year post-op mean leg VAS	0.9	0.5	0.22
Pre-op ODI^**++**^ score	28.8	29.1	0.15
2-year post-op ODI^#^ score	6.4	6.2	0.45
Pre-op mean scoliotic angle(°)	41.3	38.5	0.48
2-year post-op mean scoliotic angle(°)	9.3	21.4	0.02*
Scoliosis correction(%)	78	44	0.02*
Pre-op mean lumbar lordotic angle(°)	3.1	6	0.21
2-year post–op mean lumbar lordotic angle(°)	35.7	15.8	0.009*

^+^: VAS, Visual analog scale.

^++^: ODI, Oswestry Disability Index.

#AP, Combined anterior and posterior approach.

##:P, Posterior approach.

*:P value < 0.05.

### Radiographic outcomes

**Figure 2:**
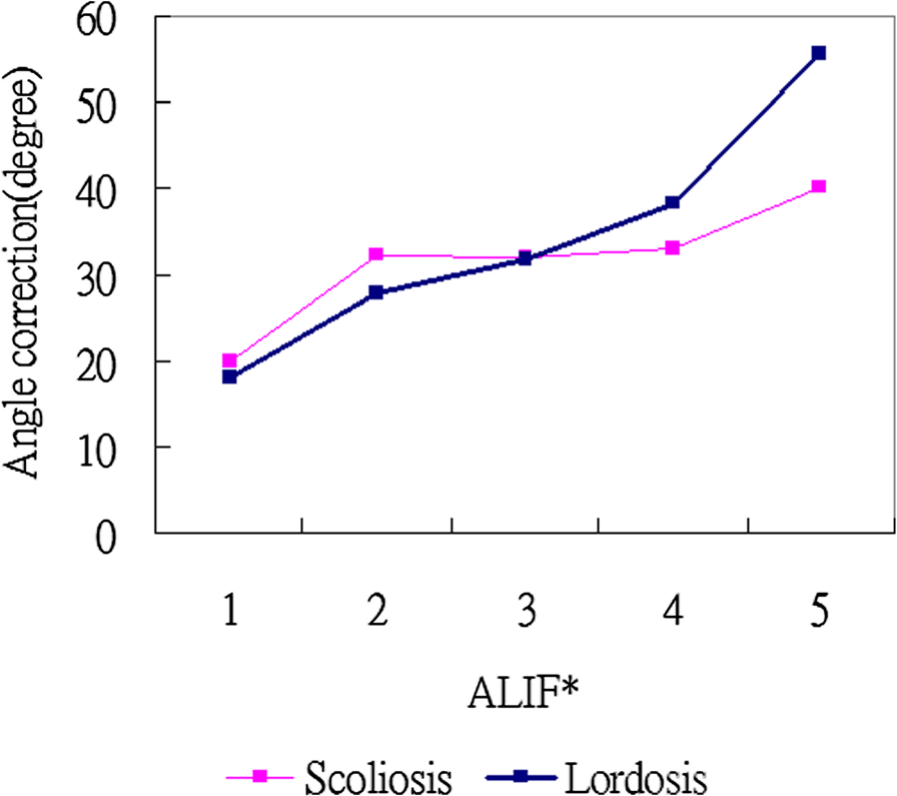
**Line chart of scoliotic angle correction compared with lordotic angle correction.**

**Figure 3:**
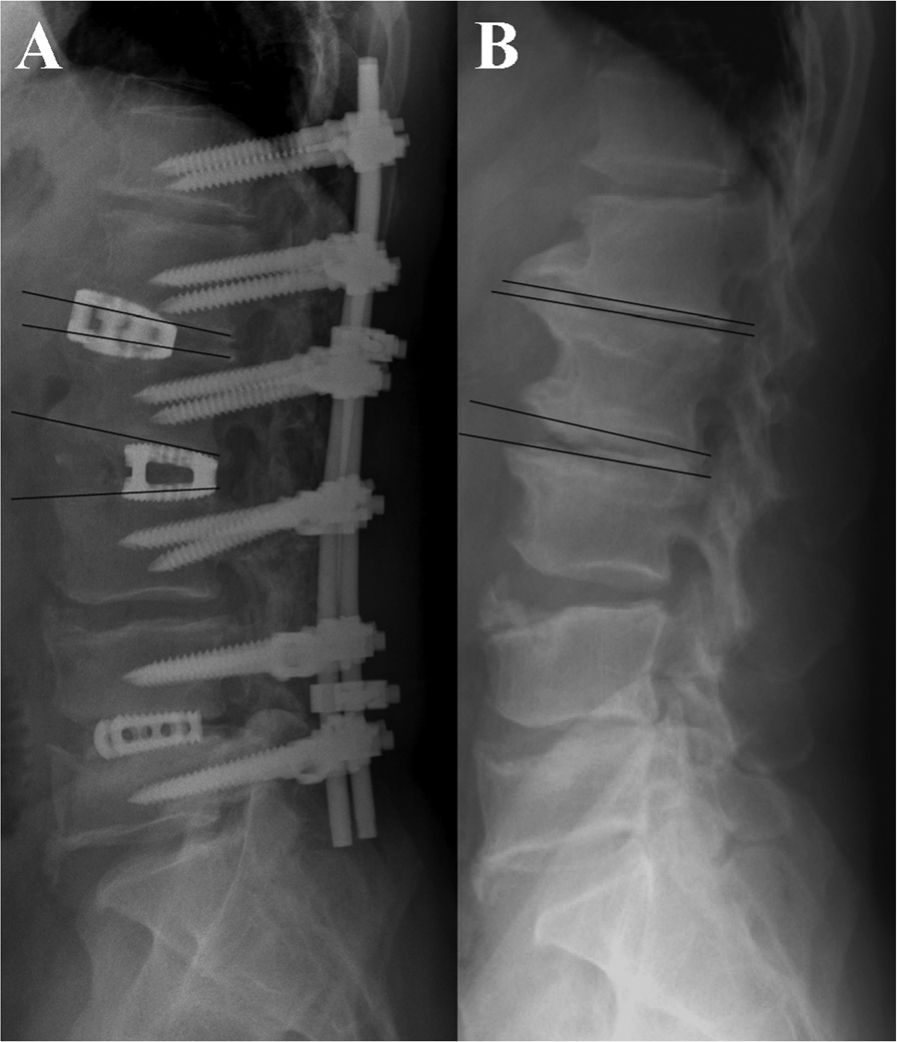
**Radiographs of postoperative lateral view (A) and preoperative lateral view (B) showing a posteriorly placed L2-3 cage provided more sagittal plane correction (2° pre-op to 19° post-op, correction 17°) than a more anterior L1-2 cage (−2° pre-op to 4° post-op, correction 6°).**

Preoperative and postoperative coronal Cobb angles and lumbar lordosis angles were compared (Table 1). The average preoperative coronal Cobb angle was 41.3° (range, 32°–85°), which decreased to 9.3° post-operatively in AP group, demonstrating a significant mean scoliosis correction of 78% (P = 0.042). The mean preoperative lumbar lordosis angle increased from 3.1° (range, kyphosis 30° to lordosis 33°) to 35.7° (range, lordosis 9° to 60°), demonstrating a mean improvement of 32.6° (P = 0.009).In P group , the average preoperative coronal Cobb angle was 38.5° (range, 32°–55°), which decreased to 21.4° post-operatively, demonstrating a significant mean scoliosis correction of 44%. The mean preoperative lumbar lordosis angle increased from 6° (range, kyphosis 25° to lordosis 25°) to 15.8° (range, lordosis 10° to 40°), demonstrating a mean improvement of 9.8°. Both in coronal and sagittal plane ,angle improvement were better in AP group than P group.As shown in Table 2, ALIF was performed for a total of 171 disc levels in the 56 patients as follows: 1-level procedure (n = 3), 2-level (n = 15), 3-level (n = 18), 4-level (n = 16), and 5-level (n = 4). As seen in Figure 2, the ALIF procedures were correlated with a higher rate of scoliosis and lordosis correction. In Figure 3 and Table 3, an ALIF cage placed in the posterior half provides more lordosis at the instrumented level, whereas a cage placed in the anterior half may not provide better sagittal plane correction. (10.9° to 6.1°; P = 0.0058). Two patients exhibited asymptomatic SynCage subsidence, and 1 patient had asymptomatic S1 screw loosening. The fusion status was decided by the senior surgeons (W-J,Chen). At the final follow-up, 36 of the 56 patients (64.3%) exhibited solid anterior fusion; 40 (71.4%), solid posterolateral fusion; 20 (35.7%), probable anterior fusion; and 16 (28.6%), probable posterolateral fusion in AP group and 39 of the 54 patients (72.2%) exhibited solid posterolateral fusion; 15 (27.8%), probable posterolateral fusion in P group. No anterior or posterolateral pseudoarthrosis was noted.

**Table 2:** **ALIF levels and angle correction**

	**Patients**	**Scoliotic angle (°)**			**Lumbar lordotic angle (°)**		
**ALIF** ^*^		**Pre-op**	**2-year F/U** ^**+**^	**Correction**	**Pre-op**	**2-year F/U**	**Correction**
1	3	22	2	20	2	20	18
2	15	39	6.8	32.2	4.1	32	27.9
3	18	41	9	32	2	33.7	31.7
4	16	44	11	33	7.8	46	38.2
5	4	62	22	40	0.5	56	55.5

^*^ALIF: Anterior lumbar interbody fusion levels.

^+^F/U: Follow up.

**Table 3:** **Position of SynCages and lordotic angle correction**

**SynCage position**	**Number**	**Mean lordotic angle correction**	**P value**
Anterior half	78 cages	6.1° (1°–12°)	
Posterior half	93 cages	10.9° (6°–24°)	0.0058

### Complications

There were no major complications such as intraoperative cerebrospinal fluid (CSF) leak, postoperative weakness, deep venous thrombosis, ureteral trauma, or injury to the peritoneal or retroperitoneal structures. Perioperative complications, which presented as postoperative superficial wound infections, occurred in 5 patients in the AP group and 7 in P group, but the symptoms subsided after debridement and antibiotic treatment. Six patients experienced transient postoperative anterior thigh numbness in the AP group, ipsilateral to the approach, in the distribution of the anterior femoral cutaneous nerve.

## Discussion

Adult degenerative scoliosis is believed to develop as a result of asymmetrical degeneration of the spine. It most commonly occurs in the lumbar spine and typically presents as pain, which is the primary complaint in 90% of the patients [7]. This axial back pain occurs most commonly due to a combination of muscle fatigue, trunk imbalance, facet arthropathy, and degenerative disc disease [20,21]. The flat-back deformity and forward sagittal imbalance have been shown to be a significant source of pain and disability in patients [22,23]. Several studies showed that radiographic parameters were correlated with clinical symptoms in adults [24-27]. Many radiographic parameters may affect functional scores in degenerative lumbar scoliosis, including the Cobb scoliosis angle in all major thoracolumbar or lumbar and lumbosacral curves, maximal intervertebral lateral olisthesis, thoracic kyphosis, thoracolumbar kyphosis, lumbar lordosis, plumbline offset from C7 to the posterosuperior corner of the S1 vertebral body, and maximal intervertebral anteroposterior olisthesis. Avraam et al. [26] reported that a loss of normal lordosis could affect health outcomes even when the sagittal balance is preserved in patients with degenerative lumbar scoliosis. Decreased lumbar lordosis and an increased lumbosacral hemicurve have led to poorer results of health status. The average curve correction in our series was 78% of maximal Cobb angle with maintenance of the correction seen at the 2-year follow-up. The mean scoliotic and lumbar lordotic angle were improved from 41.3° and 3.1° pre-operatively to 9.3° and 35.7° at the 2-year follow-up in our combined group. However , the mean scoliotic and lumbar lordotic angle were improved from 38.5° and 6° pre-operatively to 21.4° and 15.8° in our posterior approach. There were significant differences in sagittal (P = 0.009) and coronal (P = 0.02) plane correction between the combined anterior-posterior group and the posterior group. The radiographic outcome in our combined approach was superior to that via a posterior approach (Table 1).

Degenerative lumbar scoliosis with spinal stenosis has been traditionally treated with posterior decompression along with posterior spinal instrumentation and fusion. From 2002 to 2011, 1834 patients with degenerative lumbar scoliosis underwent surgery in our institution and 1778 patients were treated via a posterior-only approach. Only 56 patients received combined anterior lumbar interbody fusion and instrumented posterolateral fusion. The obvious disadvantages of using an anterior approach to the lumbar spine include retroperitoneal dissection with associated vascular manipulation, 2 major surgical procedures required, and increased operating time [8,9]. In our experience, the following are the indications for ALIF in addition to instrumented posterolateral fusion: (1) rigid or frank lumbar kyphosis; (2) anterior or lateral bridged traction vertebral osteophytes; (3) gross coronal and sagittal deformity or imbalance; and (4) severe disc space narrowing that is not identifiable when performing PLIF or TLIF. In de novo degenerative lumbar scoliosis, the curves tend to be more rigid posteriorly and, therefore, are amenable to correction via a posterior-only approach [10]. In our result ,rigid or frank lumbar kyphoscoliotic deformity secondary to idiopathic curves were difficult to correct via a posterior-only approach with scoliosis correction only 44% and lordotic angle correction only 9.8°, and better scoliosis and lordosis correction were achieved through the combined anterior and posterior approach with statistically significant. Anterior or lateral bridged traction vertebral osteophytes restrict not only graft placement but also lordosis restoration and can only be removed via an anterior approach. When performing PLIF or TLIF in osteoporotic endplate or severe disc narrowing cases, violation of the endplate always results in cage subsidence and poor angle correction.

The use of interbody grafts associated with posterior instrumentation in deformity correction surgery has gained popularity for providing anterior column structural stability, increased fusion rates, as well as enable restoration and preservation of lumbar lordosis [11-13,28]. Graft placement has traditionally been achieved through either an anterior (ALIF) or a posterior (PLIF or TLIF) approach.

Anterior lumbar interbody fusion is superior to posterior lumbar fusion because of the larger surface area available between the vertebral bodies, which facilitates the use of wider cages that can rest on a strong peripheral cortical bone on either side, thus minimizing the risk of cage subsidence, which is especially important in elderly patients with osteoporotic bones [11,17,29,30].

We found that the ALIF approach using the anterior-posterior wedge cages was effective for correcting sagittal plane deformities. An ALIF cage placed in the posterior half provides more lordosis at the instrumented level, whereas a cage placed in the anterior half may not provide better sagittal plane correction. (10.9° to 6.1°; P = 0.0058) (Figure 3, Table 3).

We have observed that the transpsoas approach leads to a high frequency of thigh numbness, pain, weakness, and dysesthesias, which are likely the result of retraction proximal to the lumbosacral plexus, and have been well described in previous anatomical studies [31]. Prior reports of lateral retroperitoneal approaches including mobilization of the psoas muscle from the lumbar spine have demonstrated high incidence (30%) of paresthesias in the thigh/groin region [32]. Knight et al. [33] reported 10% incidence of lateral femoral cutaneous nerve deficit and 3% incidence of L4 motor deficit using the lateral retroperitoneal transpsoas approach. In our study, we identified 6 patients (10.7%) with transient postoperative ipsilateral sensory deficits that had resolved before the last follow-up visit. Because these deficits were transient, we believe they were stretch or neuropraxic injuries.

## Conclusions

ALIF with SynCages and supplemental instrumented posterolateral fusion resulted in better coronal and sagittal plane correction than posterior approach in all patients and was maintained in the 2-year follow-up. The VAS and ODI scores significantly improved after the operation, and no major complications occurred.
